# Supplementary material for: The Role of Symbiotic Microorganisms, Nutrient Uptake and Rhizosphere Bacterial Community in Response of Pea (Pisum sativum L.) Genotypes to Elevated Al Concentrations in Soil
Source: Plants (Basel). 2020 Dec 18;9(12):1801. doi: 10.3390/plants9121801 (PMC7766424; doi:10.3390/plants9121801)
Supplement: Supplementary file 1 [file plants-09-01801-s001.pdf]

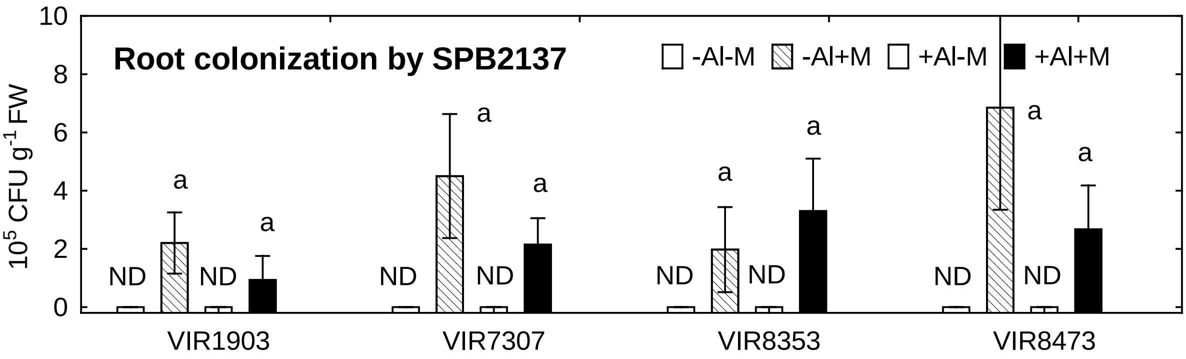

Figure S1. Root colonization by *Pseudomonas fluorescens* SPB2137 of pea genotypes VIR1903, VIR7307, VIR8353 and VIR8473 inoculated with microbial consortium and grown in neutralized or Al-supplemented soil. Treatments: -Al-M — neutralized soil with uninoculated plants, -Al+M — neutralized soil with inoculated plants, +Al-M — Al-supplemented soil with uninoculated plants, +Al+M — Al-supplemented soil with inoculated plants. Plants were inoculated with a microbial consortium consisting of *Pseudomonas fluorescens* SPB2137, *Rhizobium leguminosarum* bv. *viciae* RCAM1079 and *Glomus* sp. 1Fo. Vertical bars show standard errors. A lowercase "a" indicates no difference between treatments and pea genotypes (least significant difference test, $p < 0.05$, $n = 4$). CFU stands for colony forming units. FW stands for fresh weight. ND stands for not determined. Plants were analyzed on the 80th day after planting.

Table S1. Concentration of nutrients in the rhizosphere of pea genotypes inoculated with microbial consortium and grown in neutralized or Al-supplemented soil.

Treatments	B (ng g^{-1} DW)	Ca (μg g^{-1} DW)	Co (ng g^{-1} DW)	Cu (ng g^{-1} DW)	K (μg g^{-1} DW)
			Pea genotype VIR1903		
-Al -M	527 ± 25 ^{a}	207 ± 10 ^{ab}	53 ± 2 ^{a}	164 ± 8 ^{a}	25.4 ± 2.4 ^{a}
-Al +M	488 ± 12 ^{a}	237 ± 7 ^{b}	60 ± 6 ^{a}	161 ± 10 ^{a}	22.8 ± 1.7 ^{a}
+Al -M	782 54 ^{b}	172 ± 8 ^{a}	81 ± 8 ^{b}	170 ± 13 ^{a}	31.5 ± 1.9 ^{b}
+Al +M	*463 46 ^{a}*	**217 ± 14 ^{b}**	**107 ± 3 ^{c}**	177 ± 7 ^{a}	*25.3 ± 0.5 ^{a}*
			Pea genotype VIR7307		
-Al -M	438 ± 5 ^{a}	193 ± 13 ^{a}	71 ± 1 ^{b}	181 ± 5 ^{a}	19.4 ± 0.3 ^{a}
-Al +M	477 ± 13 ^{a}	190 ± 18 ^{a}	74 ± 5 ^{b}	191 ± 17 ^{a}	20.5 ± 2.6 ^{a}
+Al -M	523 ± 8 ^{a}	195 ± 9 ^{a}	46 ± 1 ^{a}	172 ± 2 ^{a}	17.8 ± 0.1 ^{a}
+Al +M	**680 ± 10 ^{b}**	203 ± 4 ^{a}	**103 ± 2 ^{c}**	197 ± 3 ^{a}	22.3 ± 0.3 ^{a}
			Pea genotype VIR8353		
-Al -M	538 ± 58 ^{b}	151 ± 22 ^{a}	64 ± 4 ^{c}	135 ± 9 ^{b}	20.8 ± 1.1 ^{a}
-Al +M	*407 ± 22 ^{a}*	151 ± 5 ^{a}	57 ± 1 ^{c}	136 ± 2 ^{b}	22.7 ± 0.3 ^{a}
+Al -M	447 ± 35 ^{ab}	145 ± 3 ^{a}	31 ± 2 ^{a}	99 ± 13 ^{a}	20.8 ± 1.8 ^{a}
+Al +M	477 ± 11 ^{ab}	152 ± 13 ^{a}	**45 ± 7 ^{b}**	**137 ± 19 ^{b}**	24.4 ± 2.3 ^{a}
			Pea genotype VIR8473		
-Al -M	478 ± 9 ^{a}	174 ± 5 ^{a}	66 ± 1 ^{a}	154 ± 3 ^{a}	30.0 ± 1.4 ^{a}
-Al +M	565 ± 21 ^{a}	141 ± 18 ^{a}	**100 ± 3 ^{c}**	175 ± 19 ^{a}	**35.5 ± 2.9 ^{b}**
+Al -M	512 ± 53 ^{a}	217 ± 7 ^{b}	99 ± 4 ^{c}	161 ± 5 ^{a}	36.2 ± 0.9 ^{b}
+Al +M	544 ± 61 ^{a}	**257 ± 4 ^{c}**	*84 ± 1 ^{b}*	**212 ± 16 ^{b}**	32.4 ± 0.6 ^{ab}

Plants were inoculated with a microbial consortium consisting of *Pseudomonas fluorescens* SPB2137, *Rhizobium leguminosarum* bv. *viciae* RCAM1079 and *Glomus* sp. 1Fo. Different superscript letters (a, b and c) show significant differences between treatments within a subcolumn of particular pea genotype (least significant difference test, $p < 0.05$, $n = 4$). Values in bold or italicized indicate significant positive or negative effect of the microbial consortium, respectively. Data are means ± SE. DW stands for dry weight. Plants were analyzed on the 80th day after planting.

Table S2. Concentration of nutrients in shoots of pea genotypes inoculated with microbial consortium and grown in neutralized or Al-supplemented soil.

Treatments	B (ng g^{-1} DW)	Ca (µg g^{-1} DW)	Co (ng g^{-1} DW)	Cu (ng g^{-1} DW)
		Pea genotype VIR1903		
-Al -M	10.7 ± 0.4 ^{a}	30.8 ± 1.2 ^{a}	4.9 ± 0.1 ^{a}	20.2 ± 0.6 ^{a}
-Al +M	**14.1 ± 0.5** ^{b}	33.9 ± 0.7 ^{a}	5.0 ± 0.1 ^{a}	**22.8 ± 0.4** ^{b}
+Al -M	14.4 ± 0.5 ^{b}	31.2 ± 0.6 ^{a}	6.0 ± 0.1 ^{b}	19.1 ± 0.3 ^{a}
+Al +M	13.8 ± 0.9 ^{b}	34.4 ± 1.4 ^{a}	6.3 ± 0.2 ^{b}	**22.1 ± 0.8** ^{b}
		Pea genotype VIR7307		
-Al -M	8.5 ± 0.4 ^{a}	31.8 ± 0.5 ^{a}	4.6 ± 0.1 ^{a}	15.4 ± 0.2 ^{a}
-Al +M	7.3 ± 0.4 ^{a}	27.9 ± 1.7 ^{a}	5.0 ± 0.2 ^{a}	**20.8 ± 0.9** ^{c}
+Al -M	12.5 ± 0.6 ^{b}	31.0 ± 0.8 ^{a}	5.9 ± 0.1 ^{b}	17.3 ± 0.4 ^{b}
+Al +M	12.9 ± 0.6 ^{b}	31.8 ± 0.9 ^{a}	6.0 ± 0.1 ^{b}	17.4 ± 0.4 ^{b}
		Pea genotype VIR8353		
-Al -M	23.4 ± 1.1 ^{a}	58.9 ± 1.4 ^{a}	7.4 ± 0.1 ^{a}	30.0 ± 0.5 ^{ab}
-Al +M	22.9 ± 0.2 ^{a}	62.8 ± 0.7 ^{a}	7.3 ± 0.1 ^{a}	31.5 ± 0.3 ^{b}
+Al -M	31.0 ± 0.5 ^{b}	62.9 ± 0.8 ^{a}	8.9 ± 0.1 ^{b}	28.8 ± 0.3 ^{a}
+Al +M	29.0 ± 0.2 ^{b}	60.4 ± 0.6 ^{a}	8.8 ± 0.1 ^{b}	28.7 ± 0.3 ^{a}
		Pea genotype VIR8473		
-Al -M	14.1 ± 0.4 ^{a}	29.9 ± 0.2 ^{ab}	6.3 ± 0.1 ^{a}	29.9 ± 1.7 ^{a}
-Al +M	**18.6 ± 2.1** ^{b}	31.1 ± 4.6 ^{ab}	**9.0 ± 1.0** ^{c}	**38.0 ± 0.1** ^{b}
+Al -M	16.7 ± 0.4 ^{b}	27.5 ± 0.7 ^{a}	7.0 ± 0.1 ^{ab}	30.6 ± 0.5 ^{a}
+Al +M	18.7 ± 0.3 ^{b}	**33.6 ± 0.8** ^{b}	7.9 ± 0.1 ^{b}	31.2 ± 0.2 ^{a}

Plants were inoculated with a microbial consortium consisting of *Pseudomonas fluorescens* SPB2137, *Rhizobium leguminosarum* bv. *viciae* RCAM1079 and *Glomus* sp. 1Fo. Different superscript letters (a, b and c) show significant differences between treatments within a subcolumn of particular pea genotype (least significant difference test, $p < 0.05$, $n = 4$). Values in bold or italicized indicate significant positive or negative effect of the microbial consortium, respectively. Data are means ± SE. DW stands for dry weight. Plants were analyzed on the 80th day after planting.

Table S3. Concentration of nutrients in seeds of pea genotypes inoculated with microbial consortium and grown in neutralized or Al-supplemented soil.

Treatments	**B** (ng g^{-1} DW)	**Ca** (μg g^{-1} DW)	**Co** (ng g^{-1} DW)	**Cu** (ng g^{-1} DW)	**Fe** (ng g^{-1} DW)	**K** (μg g^{-1} DW)	**Mg** (μg g^{-1} DW)	**Mn** (ng g^{-1} DW)	**Mo** (ng g^{-1} DW)	**Ni** (ng g^{-1} DW)	**P** (μg g^{-1} DW)	**S** (μg g^{-1} DW)	**Zn** (ng g^{-1} DW)
						Pea genotype VIR1903							
-Al -M	2.4 ± 0.3 ^{a}	0.9 ± 0.1 ^{a}	0.7 ± 0.1 ^{a}	43 ± 4 ^{a}	144 ± 8 ^{a}	9.3 ± 0.4 ^{a}	1.3 ± 0.1 ^{a}	13 ± 1 ^{a}	1.2 ± 0.1 ^{ab}	1.3 ± 0.3 ^{a}	36 ± 2 ^{a}	0.83 ± 0.03 ^{a}	38 ± 3 ^{a}
-Al +M	**4.7 ± 0.7 ^{b}**	0.9 ± 0.2 ^{a}	0.7 ± 0.1 ^{a}	42 ± 4 ^{a}	184 ± 37 ^{a}	9.3 ± 0.6 ^{a}	1.3 ± 0.1 ^{a}	13 ± 2 ^{a}	1.5 ± 0.3 ^{b}	1.6 ± 0.4 ^{a}	37 ± 3 ^{a}	0.85 ± 0.03 ^{a}	**62 ± 3 ^{b}**
+Al -M	2.6 ± 0.3 ^{a}	0.9 ± 0.1 ^{a}	0.4 ± 0.1 ^{a}	39 ± 2 ^{a}	147 ± 16 ^{a}	8.9 ± 0.3 ^{a}	1.2 ± 0.1 ^{a}	13 ± 1 ^{a}	0.6 ± 0.1 ^{a}	1.0 ± 0.3 ^{a}	34 ± 1 ^{a}	0.77 ± 0.02 ^{a}	38 ± 1 ^{a}
+Al +M	3.9 ± 0.4 ^{ab}	0.9 ± 0.1 ^{a}	0.6 ± 0.1 ^{a}	40 ± 4 ^{a}	154 ± 8 ^{a}	9.2 ± 0.3 ^{a}	1.2 ± 0.1 ^{a}	14 ± 1 ^{a}	0.6 ± 0.1 ^{a}	0.9 ± 0.1 ^{a}	36 ± 4 ^{a}	0.89 ± 0.02 ^{a}	43 ± 2 ^{a}
						Pea genotype VIR7307							
-Al -M	3.3 ± 0.3 ^{a}	1.1 ± 0.1 ^{a}	0.9 ± 0.1 ^{a}	36 ± 4 ^{a}	117 ± 11 ^{a}	11.7 ± 1.1 ^{a}	1.7 ± 0.1 ^{a}	12 ± 1 ^{a}	2.1 ± 0.2 ^{b}	1.7 ± 0.2 ^{a}	35 ± 4 ^{a}	1.21 ± 0.10 ^{a}	36 ± 2 ^{a}
-Al +M	4.1 ± 0.3 ^{ab}	1.2 ± 0.1 ^{ab}	1.3 ± 0.1 ^{a}	40 ± 3 ^{a}	172 ± 21 ^{ab}	13.7 ± 0.9 ^{ab}	2.0 ± 0.1 ^{a}	15 ± 1 ^{a}	2.3 ± 0.2 ^{b}	**4.6 ± 0.9 ^{b}**	39 ± 3 ^{a}	1.37 ± 0.11 ^{a}	46 ± 4 ^{a}
+Al -M	4.9 ± 0.8 ^{b}	1.5 ± 0.2 ^{bc}	1.3 ± 0.1 ^{a}	42 ± 7 ^{a}	191 ± 30 ^{b}	15.0 ± 2.2 ^{b}	1.7 ± 0.3 ^{a}	23 ± 3 ^{b}	1.0 ± 0.2 ^{a}	2.3 ± 0.5 ^{a}	40 ± 7 ^{a}	1.24 ± 0.16 ^{a}	70 ± 11 ^{b}
+Al +M	4.4 ± 0.4 ^{ab}	1.8 ± 0.2 ^{c}	1.3 ± 0.2 ^{a}	38 ± 3 ^{a}	147 ± 26 ^{ab}	13.6 ± 1.0 ^{ab}	2.0 ± 0.1 ^{a}	21 ± 2 ^{b}	0.9 ± 0.1 ^{a}	2.8 ± 0.8 ^{a}	36 ± 2 ^{a}	1.16 ± 0.08 ^{a}	63 ± 7 ^{b}
						Pea genotype VIR8353							
-Al -M	3.3 ± 0.9 ^{a}	1.9 ± 0.3 ^{ab}	1.0 ± 0.1 ^{a}	43 ± 3 ^{a}	136 ± 15 ^{a}	9.4 ± 0.8 ^{a}	1.6 ± 0.1 ^{a}	20 ± 3 ^{a}	2.1 ± 0.2 ^{b}	2.3 ± 0.6 ^{a}	48 ± 5 ^{a}	1.04 ± 0.08 ^{a}	46 ± 5 ^{a}
-Al +M	2.9 ± 0.4 ^{a}	1.9 ± 0.2 ^{ab}	**1.5 ± 0.2 ^{b}**	**67 ± 6 ^{b}**	**201 ± 21 ^{b}**	**12.6 ± 1.3 ^{b}**	**2.2 ± 0.2 ^{b}**	19 ± 2 ^{a}	2.1 ± 0.2 ^{b}	2.1 ± 0.2 ^{a}	**71 ± 5 ^{b}**	**1.44 ± 0.12 ^{b}**	56 ± 3 ^{ab}
+Al -M	2.9 ± 0.3 ^{a}	1.7 ± 0.1 ^{a}	1.2 ± 0.1 ^{ab}	53 ± 4 ^{a}	216 ± 14 ^{b}	10.2 ± 0.4 ^{b}	1.9 ± 0.1 ^{ab}	22 ± 1 ^{ab}	0.9 ± 0.1 ^{a}	2.5 ± 0.3 ^{a}	54 ± 5 ^{a}	1.15 ± 0.04 ^{a}	65 ± 5 ^{b}
+Al +M	**4.5 ± 0.6 ^{b}**	**2.2 ± 0.1 ^{b}**	**2.0 ± 0.3 ^{c}**	**73 ± 9 ^{b}**	**284 ± 21 ^{c}**	**15.1 ± 1.9 ^{c}**	**2.7 ± 0.3 ^{c}**	26 ± 2 ^{b}	1.5 ± 0.3 ^{a}	3.3 ± 0.4 ^{a}	**78 ± 10 ^{b}**	**1.61 ± 0.16 ^{b}**	**81 ± 6 ^{c}**
						Pea genotype VIR8473							
-Al -M	3.0 ± 0.8 ^{a}	1.1 ± 0.1 ^{a}	0.7 ± 0.1 ^{a}	50 ± 1 ^{a}	134 ± 12 ^{a}	10.3 ± 0.4 ^{a}	1.4 ± 0.1 ^{a}	10 ± 1 ^{a}	3.2 ± 0.1 ^{b}	1.0 ± 0.1 ^{a}	49 ± 1 ^{a}	0.97 ± 0.03 ^{a}	40 ± 2 ^{a}
-Al +M	3.7 ± 0.5 ^{ab}	1.1 ± 0.1 ^{a}	**1.9 ± 0.3 ^{b}**	55 ± 5 ^{a}	148 ± 5 ^{a}	12.5 ± 1.1 ^{a}	1.6 ± 0.1 ^{a}	**21 ± 3 ^{c}**	3.5 ± 0.2 ^{b}	1.6 ± 0.2 ^{a}	53 ± 5 ^{a}	1.16 ± 0.08 ^{ab}	**61 ± 6 ^{b}**
+Al -M	3.5 ± 0.5 ^{a}	1.1 ± 0.1 ^{a}	0.4 ± 0.2 ^{a}	56 ± 4 ^{a}	128 ± 9 ^{a}	11.3 ± 0.2 ^{a}	1.6 ± 0.1 ^{a}	14 ± 1 ^{ab}	1.4 ± 0.2 ^{a}	5.5 ± 0.6 ^{b}	55 ± 3 ^{a}	0.96 ± 0.01 ^{a}	56 ± 4 ^{b}
+Al +M	**5.4 ± 0.7 ^{b}**	**2.0 ± 0.1 ^{b}**	**1.9 ± 0.1 ^{b}**	63 ± 6 ^{a}	**204 ± 25 ^{b}**	**16.5 ± 1.4 ^{b}**	**2.3 ± 0.2 ^{b}**	18 ± 1 ^{bc}	1.7 ± 0.4 ^{a}	6.3 ± 0.6 ^{b}	61 ± 5 ^{a}	**1.21 ± 0.08 ^{b}**	51 ± 6 ^{ab}

Plants were inoculated with a microbial consortium consisting of *Pseudomonas fluorescens* SPB2137, *Rhizobium leguminosarum* bv. *viciae* RCAM1079 and *Glomus* sp. 1Fo. Different superscript letters (a, b and c) show significant differences between treatments within a subcolumn of particular pea genotype (least significant difference test, $p < 0.05$, $n = 4$). Values in bold indicate significant positive effect of the microbial consortium. Data are means ± SE. DW stands for dry weight. Plants were analyzed on the 80th day after planting.

Table S4. Alpha diversity indices for the rhizosphere prokaryotic microbiomes of pea genotypes VIR1903, VIR7307, VIR8353 and VIR8473 inoculated with microbial consortium and grown in neutralized or Al-supplemented soil.

Treatments	Obs_phylotypes	Chao1	Faith_PD	Shannon	Simpson
		Pea genotype VIR1903			
-Al -M	1615 ± 48	1741± 59	288 ± 9	9.49 ± 0.05	0.997 ± 0.0
-Al +M	1609 ± 11	1719± 12	280 ± 4	9.52 ± 0.04	0.997 ± 0.0
+Al -M	1583 ± 54	1681 ± 71	285 ± 11	9.50 ± 0.06	0.997 ± 0.0
+Al +M	1488 ± 27	1538 ± 34	260 ± 7	9.55 ± 0.03	0.997 ± 0.0
		Pea genotype VIR7307			
-Al -M	1594 ± 50	1681 ± 58	260 ± 6	9.60 ± 0.07	0.997 ± 0.0
-Al +M	1628 ± 31	1765 ± 54	275 ± 5	9.21 ± 0.04	0.991 ± 0.0
+Al -M	1613 ± 51	1747 ± 76	280 ± 6	9.24 ± 0.06	0.991 ± 0.0
+Al +M	1457 ± 154	1536 ± 177	254 ± 26	9.23 ± 0.14	0.993 ± 0.0
		Pea genotype VIR8353			
-Al -M	1588 ± 65	1755 ± 88	264 ± 11	8.90 ± 0.13	0.989 ± 0.0
-Al +M	1675 ± 42	1879 ± 69	281 ± 9	9.13 ± 0.07	0.993 ± 0.0
+Al -M	1578 ± 44	1752 ± 58	270 ± 4	9.02 ± 0.02	0.992 ± 0.0
+Al +M	1669 ± 79	1928 ± 127	292 ± 11	8.81 ± 0.17	0.987 ± 0.0
		Pea genotype VIR8473			
-Al -M	1295 ± 72	1338 ± 78	219 ± 11	9.29 ± 0.10	0.995 ± 0.0
-Al +M	1580 ± 150	1698 ± 193	253 ± 16	9.50 ± 0.11	0.996 ± 0.0
+Al -M	1385 ± 39	1445 ± 44	233 ± 4	9.34 ± 0.06	0.995 ± 0.0
+Al +M	1412 ± 58	1500 ± 82	250 ± 8	9.00 ± 0.11	0.992 ± 0.0

Treatments: -Al-M — neutralized soil with uninoculated plants, -Al+M — neutralized soil with inoculated plants, +Al-M — Al-supplemented soil with uninoculated plants, +Al+M — Al-supplemented soil with inoculated plants. Plants were inoculated with a microbial consortium consisting of *Pseudomonas fluorescens* SPB2137, *Rhizobium leguminosarum* bv. *viciae* RCAM1079 and *Glomus* sp. 1Fo. Data are means ± SE. Plants were analyzed on the 80th day after planting.
